# Changing trends in blood transfusion in children and neonates admitted in Kilifi District Hospital, Kenya

**DOI:** 10.1186/1475-2875-9-307

**Published:** 2010-10-30

**Authors:** Rosalon Pedro, Samuel Akech, Kathryn Maitland

**Affiliations:** 1 Kenya Medical Research Institute-Wellcome Trust Programme, PO Box 230, Kilifi, Kenya; 2 Centro de Investigação em Saúde em Angola (CISA), Caxito, Angola; 3 Department of Paediatrics and Wellcome Trust Centre for Clinical Tropical Medicine, Faculty of Medicine, Imperial College, Norfolk Place, London, W2 1PG, UK

## Abstract

**Background:**

Severe anaemia is a common cause for hospitalization in children in sub-Saharan Africa. Malaria plays an important aetiological role, resulting in a substantial burden of paediatric transfusion in hospitals. A decline in malaria and paediatric admissions to the Kilifi District Hospital has been reported recently. This study aimed to investigate whether this trend affected clinical burden, clinical severity of anaemia and requirements for paediatric transfusion.

**Methods:**

Eight-year retrospective review of paediatric admissions to Kilifi District Hospital, Kenya describing the frequency of moderate and severe anaemia, blood transfusion and case fatality over time. Definitions for severe anaemia were Hb <8 g/dl for newborns and <5 g/dl for other age groups and for moderate anaemia was Hb 8 to <11 g/dl for newborns and 5 to <9.3 g/dl for other age groups. Life threatening anaemia was defined as severe anaemia (Hb <5 g/dl) complicated by either deep breathing or prostration or profound anaemia (Hb <4 g/dl) alone.

**Results:**

Of the 35,139 admissions 13,037 (37%) had moderate anaemia and 2,265 (6%) had severe anaemia; respiratory distress complicated 35% of cases with Hb <5 g/dl. Concurrent with the decline in malaria there was a marked decline in the prevalence of severe anaemia between 2002 (8%) and 2009 (< 4%) (chi^2 ^for trend = 134, P < 0.0001). The number and proportion of admissions transfused also declined significantly over this time (chi2 for trend = 152, P < 0.0001). Of the 2,265 children with severe anaemia 191 (8%) died. Case fatality remained unchanged during this period (P < 0.26) and was largely explained by the unchanged proportion with life-threatening anaemia, present in 58-65% of cases throughout the study period.

**Conclusion:**

The impact of reduced malaria transmission on child morbidity has positive public benefits on the demand and use of blood for paediatric transfusion. Despite an overall reduction in paediatric transfusion requirement, case fatality of severe anaemia remained unchanged over this decade. Further research is required to improve outcome from severe anaemia, particularly in the high-risk group with life threatening features.

## Background

In sub-Saharan Africa severe anaemia is a leading cause of paediatric admission to hospital. The community prevalence of anaemia in African children, defined as a haemoglobin (Hb) <11 g/dl, ranges from 49% to 89% and severe anaemia (variously defined as Hb <5 g/dl, <6 g/dl or Hb <7 g/dl) affects between 1%- 6% of children, with infants being the most vulnerable age group[1]. In hospitalized children the prevalence of severe anaemia ranges from 8 - 29%[2,3], resulting in a high demand for blood transfusion, thus in malaria-endemic Africa paediatric transfusion accounts for up to 70% of all transfusions prescribed[4].

The aetiology of anaemia is frequently multifactorial[5], commonly associated with *Plasmodium falciparum *malaria infection[6] and nutritional factors in children admitted to hospital[2,7]. The relative risk of in-hospital mortality for children with severe anaemia is almost twice that of their less anaemic counterparts[2]. Despite the provision of blood transfusion, the case fatality of children with severe anaemia remains high. Over 50% of deaths occurring within hours of admission with a number occurring in children awaiting urgent transfusion[7,8]. Blood transfusion is the definitive and life-saving treatment for acute severe anaemia, resulting in substantial benefit in children with profound anaemia (Hb < 4 g/dl) and children with Hb 4-6 g/dl with severe complications (prostration or deep breathing)[8,9]. However, the majority of transfusions are received by children with severe anaemia (Hb 4-6 g/dl) without life-threatening signs for whom the benefits of urgent transfusion are unproven[10-12]. WHO recommends avoidance of blood transfusion in this group to protect supplies of blood and reduce the risk of adverse reactions and transfusion transmitted infections[4]. Post-discharge morbidity and mortality are important considerations in this group but there are few data on the cumulative incidence of poor outcomes over the longer-term [13].

Despite these guidelines being in place for many years in Africa, up to 50% of hospitalized children in malaria endemic receive a blood transfusion[13]. Malaria still plays an important role in the onset of severe anaemia, placing a limitation on the ability to reduce the need for paediatric transfusion in hospitals in malaria endemic areas. In Kilifi District Hospital (KDH), Kenya's coast, the policy of restrictive blood transfusion practice has been applied since the late 1990's. English *et al *evaluated the practice between 1998 and 2000 and found that 13% of all paediatric admissions (excluding newborns) were transfused[8]. Malaria and severe malnutrition were the main primary diagnoses. In recent years, a decline in malaria and paediatric admissions to the hospital has been reported[14]. This review aimed to investigate whether this trend also affected transfusion practice and requirements for paediatric and neonatal transfusion.

## Methods

A retrospective study of hospital admissions to address the following specific objectives. First, to describe the proportions of anaemia and blood transfusions secondary to malaria and describe the changes over time. Secondly, to examine compliance with the threshold for blood transfusion, established by the World Health Organization (WHO) current transfusion guidelines, and finally, to determine in-hospital survival. Retrospective data review of the clinical surveillance database covering all child and neonatal admissions to Kilifi District Hospital (KDH), Kenya, over an 8-year period, from January 2002 to December 2009. Clinical data are routinely collected prospectively at admission and directly entered into a Filemaker Pro^® ^database, which is password-protected and has limited access. Data on the following were retrieved; age, sex, diagnosis, falciparum malaria parasitaemia, haemoglobin (Hb) level, respiratory distress, level of consciousness, nutritional status, capillary refill > 2 seconds (as a sign of hypovolvaemic shock) together with data on blood transfusion, length of stay and mortality as outcome variables.

Kilifi District is one of the poorest area in Kenya, where malaria is endemic, with 40% of children under five years presenting anthropometric measures of malnutrition, half with biochemical markers of iron deficiency, 14% of children have a sickle cell trait and nearly 60% have α+ thalassaemia deletion genotypes [11].

### Analysis

Proportions of transfusions due to malaria and other diagnoses were calculated for each year and the proportions compared over time. Since transfusion thresholds are different for neonates, we analysed the trends in neonates separately from those of older children. Children outside the neonatal period (≥ 1 month) were further sub-grouped into infants (1-12 months), 1-4 years, and older then 5 years since the prevalence of severe malaria anaemia varies considerably over these age groups. The children were further categorised by their clinical severity features.

Severe anaemia was defined as Hb <8 g/dl for neonates and <5 g/dl for other age groups; profound anaemia in non-neonatal groups was defined as Hb <4 g/dl. As defined by WHO criteria for anaemia: moderate anaemia was a Hb 8 - <11 g/dl for newborns and 5 - <9.3 g/dl for other age groups; .mild or no anaemia as Hb >11 g/dl for newborns and > 9.3 g/dl for other age groups. Respiratory distress was defined as either the presence of deep breathing (a clinical feature of metabolic acidosis) or indrawing. Nutritional status was measured by the parameter birth weight or gestational age for neonates (severe malnutrition was equivalent to prematurity or small for gestational age). For the other age groups severe malnutrition was defined as either mid upper arm circumference (MUAC) <11 cm and/or presence of oedema (kwashiorkor). Impaired consciousness indicated the presence of prostration (inability to sit unsupported if > 9 months or inability to breast feed if ≤ 9 months). Life-threatening anaemia was defined as either profound anaemia (Hb <4 g/dl) irrespective of clinical severity or severe anaemia (Hb <5 g/dl) complicated by deep breathing or prostration. Stata version 11 (Stata corporation, Texas, United States) was used for data analysis for descriptive statistics.

## Results

**Figure 1:**
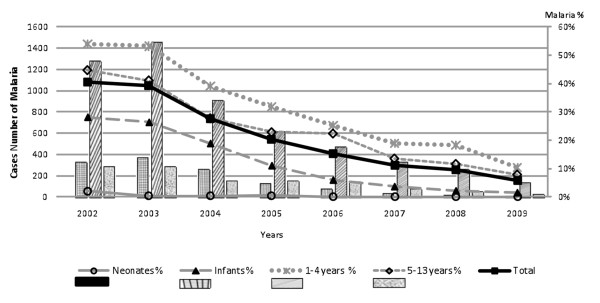
**Paediatric admissions with malaria parasitaemia 2002-2009**.

Over the eight year period 36,621 children <13 years were admitted. Admission haemoglobin (Hb) was available for 35,139 and missing in 1482 (3.9%). Moderate anaemia occured in 13,037 (37%) and 2,265 (6%) had severe anaemia (Table 1). Severe anaemia was commonest in children 1-12 years (8%) and less prevalent in newborns, with only 91 (2%) cases being affected. Respiratory distress complicated 35% of cases with Hb <5 g/dl, and was more prevalent in infants and newborn. Concomitant malaria parasitaemia, rarely present in neonates, was common in all other age groups but its prevalence was more closely tied to the year of study than age. Over the study period there was a significant decline in malaria across all ages groups (Figure 1) (chi^2 ^test for trend = 2440, P < 0.0001), which is covered in more detail in a previous report[14]. Primary and secondary diagnoses were recorded by clinicians at discharge. The most common discharge diagnoses were anaemia (76%), *P. falciparum *malaria (46%) and all types of malnutrition with 13% (Table 2).

**Table 1:** Main clinical characteristics of admissions by age group

FEATURES	Age Groups				
	**Neonates**	**Infant**	**Children 1-4 years**	**Children 5-13 years**	**Total**

**N (%)**	5114 (14)	10167 (28)	16395 (45)	4945 (14)	36621

**Age median**	3 days	6.5 months	2 years	7.5 years	1.4 years

**Male **n (%)	2987 (58)	5787 (57)	9122 (56)	2744 (55)	20640 (56)

**Malaria parasitemia **(%)	18 (0.4)	1284 (13)	5512 (35)	1224 (26)	8038 (23)

**Transfused **(%)	347 (7)	642 (7)	1338 (9)	395 (8)	2722 (8)

**Not transfused **(%)	4570 (93)	9073 (93)	14307 (91)	4335 (92)	32285 (92)

Transfusion Missing value	197	452	750	215	1614

**Mild or without anaemia **(%)	4335 (91)	5127 (53)	7378 (47)	2997 (63)	19837 (56)

**Moderate anaemia **(%)	359 (8)	4087 (42)	7177 (45)	1414 (30)	13037 (37)

**Severe anaemia **(%)	91 (2)	526 (5)	1279 (8)	369 (8)	2265 (6)

Haemoglobin value missing	329	427	561	165	1482

**Severe anaemia + Respiratory distress **(%)	38 (42)	261 (50)	413 (32)	72 (20)	784 (35)

**Severe anaemia case fatality **(%)	28 (31)	35 (7)	91 (7)	37 (10)	191 (8)

**Table 2:** Clinicians primary and secondary discharge diagnosis of children with severe anaemia

Diagnosis	Primary	Secondary	Total (%)
Anaemia	760	960	1720 (76)

*Plasmodium falciparum *malaria	730	316	1046 (46)

Severe malnutrition	218	66	284 (13)

Lower respiratory tract infection	118	79	197 (9)

Sickle cell disease	148	33	181 (8)

Septicaemia/Sepsis	66	55	121 (5)

Gastroenteritis	33	23	56 (2)

Encephalopathy	16	17	33 (1)

Neonatal jaundice	23	10	33 (1)

HIV exposed or infected	19	39	58 (3)

Other	124	151	275 (12)

Not recorded	10		10 (0.4)

Total	2265	1749	

**Figure 2:**
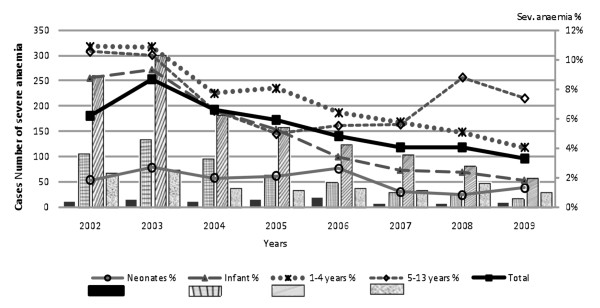
**Cases with severe anaemia by age group and year of admission**.

Concurrent with the decline in malaria there was a marked decline in the prevalence of severe anaemia (Figure 2) from 2002 to 2009 (chi^2 ^for trend = 134, P < 0.0001). Overall, 8% of admissions in 2002 had severe anaemia which decreased to < 4% by 2009. The effect was the most dramatic in infants and young children where proportions with severe anaemia declined from 10% of admissions to 2%. A steep decline was present in most age groups except children 5-12 years who showed an initial steep decline in prevalence in the years 2002-2005, which plateaued then rose again in 2008.

### Transfusion

**Figure 3:**
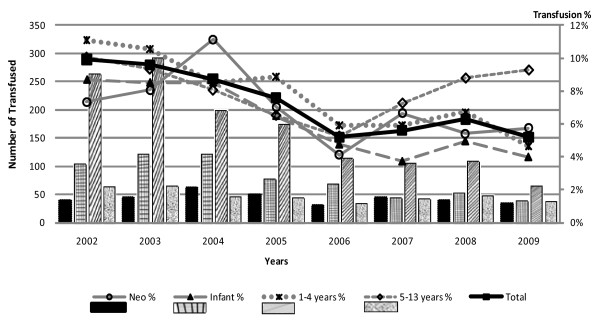
**Numbers of children receiving a blood transfusion by year and age group**.

Two thousand seven hundred and twenty two children received a transfusion (7.4%). The majority of transfusions, 55% (1,490), were received by children with severe anaemia, 29% (789) were received by children admitted with moderate anaemia (Hb 5.0-9.3 g/dl) and 14% (375) received by children who had mild or no anaemia (Hb >9.3 g/dl) at admission. Two percent of transfusions were given to children whose Hb values were not recorded on admission. These relative proportions were constant throughout the period of the study. The number and proportion of admissions transfused declined significantly over time (chi^2 ^for trend = 152, P < 0.0001) mirroring the reduction in severe anaemia (Figure 3). In the 5-12 year age group, there was an increase in transfusions in 2007-2009 which was largely accounted for by the increase in severe anaemia.

### Clinical severity of anaemia

Of the 2265 children with severe anaemia 191 (8%) died. The highest case fatality was in the neonatal group (31%) (Table 1). There was little difference in mortality in children with severe anaemia who received a transfusion, 9% (129/1490), and those who were not transfused, 8% (59/737) (Table 3). Admission features of children with severe anaemia were compared in fatal and non-fatal cases (Table 3). Respiratory distress was common 784 (24%) and associated with an elevated mortality of 15% (115/784 patients); children < 1 year being most affected. Deep 'acidotic' breathing had a higher case fatality (20%) than indrawing (14%). Prostration was present in 414 (20%) and, of these, 20% (81/414 patients) died. Shock (defined by capillary refilling time> 2 seconds) complicated 460 (20%) cases and 18% (81/460 patients) died. Severe malnutrition, incorporating both marasmus (or low gestational age for newborns) and kwashiorkor complicated 18% (413/2,265 patients) of cases of severe anaemia with an overall case fatality of 17% (70/413). Both age and type of malnutrition (kwashiorkor) affected outcome. Malaria parasitaemia was most common in infants with severe anaemia, 1,113 (49%), but rare in neonates. Malaria parasitaemia was associated with a lower case fatality 7% (74/1,118 patients) compared children with any other complication or co-morbidity.

**Table 3:** Distribution of severe anaemia and fatality by severity features and age group.

CLINICAL FEATURES	Neonates		Infants		Children 1-4 yrs		Children 5-13 yrs		Total	
	
	n (%)	CF (%)	n (%)	CF (%)	n (%)	CF (%)	n (%)	CF (%)	N (%)	CF (%)
**N**	**91**		**526**		**1279**		**369**		**2265**	

Age median [IQR]	4 days [2-10]		8 mths [6-10]		2 yrs [1.5-3]		7.5 yrs [6-9.5]		1.9 yrs [0.9-3.6]	

Respiratory Distress	38 (42)	12 (32)	261 (50)	27 (10)	413 (32)	59 (14)	72 (20)	17 (24)	784 (24)	115 (15)

Deep Breathing	21 (23)	10 (48)	118 (22)	19 (16)	261 (20)	47 (18)	41 (11)	11 (27)	441 (19)	87 (20)

Indrawing	31 (34)	10 (32)	208 (40)	20 (10)	253 (20)	33 (13)	52 (14)	13 (25)	544 (24)	76 (14)

Capillary refill >2 s	18 (20)	7 (39)	108 (21)	16 (15)	279 (22)	39 (14)	55 (15)	19 (35)	460 (20)	81 (18)

Severe malnutrition	36 (40)	16 (44)	98 (19)	10 (10)	147 (19)	35 (14)	32 (9)	9 (28)	413 (18)	70 (17)

Prostration	16 (18)	6 (38)	85 (16)	18 (21)	277 (22)	48 (17)	36 (10)	9 (25)	414 (18)	81 (20)

Malaria parasites	2 (2)	0 (0)	288 (55)	16 (6)	740 (58)	52 (7)	83 (22)	6 (7)	1113 (49)	74 (7)

**Transfused**	44 (48)	13 (30)	346 (66)	28 (8)	842 (66)	63 (7)	258 (70)	25 (10)	1490 (66)	129 (9)

**Not transfused**	47 (52)	15 (32)	164 (31)	6 (4)	423 (33)	27 (6)	103 (28)	11 (11)	737 (33)	59 (8)

No transfusion status	0		16 (3)	1 (6)	14 (1)	1 (7)	8 (2)	1 (13)	38 (2)	3 (8)

CF = Case Fatality

IQR = Interquartile range

Respiratory Distress = Deep Breathing or Indrawing

Severe malnutrition = mid-upper arm circumference <11 cm or kwashiorkor or premature or small for gestational age <2.5 Kg for neonatal group.

### Mortality over time

**Figure 4:**
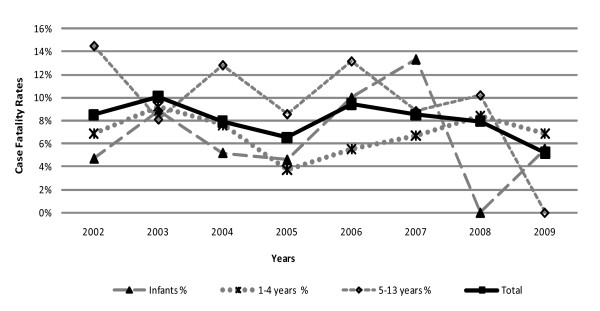
**Cases Fatality of severe anaemia by age group and year**.

Data were examined to determine whether the decline in malaria, severe anaemia and requirements for transfusion had any bearing on the case fatality rates of severe anaemia (Figure 2). The absolute number of deaths from severe anaemia declined due to a reduction in case burden however, case fatality remained unchanged during this period with an overall mortality of 8% (P < 0.26) (Figure 4). The data were explored to determine whether this could be explained by changes in the proportions with severe life threatening anaemia. Children in the high-risk group (life threatening anaemia) were selected to study trends over time (Table 4). Whilst the absolute number cases of severe anaemia fell sharply over the years the proportion of those at high risk remained unchanged being present in 58-65% of children with Hb < 5 g/dl (severe anaemia). Also notable was that the proportion of life-threatening cases did not vary with age group - with all post neonatal groups having > 60% of cases complicated by severity features.

**Table 4:** Severe anaemia complicated by Deep breathing, Prostration or Profound anaemia

Year	Neonates		Infants		Children 1-4 yrs		Children 5-13 yrs		Total	
	
	n	D.P.P (%)	n	D.P.P(%)	n	D.P.P (%)	n	D.P.P (%)	N	D.P.P (%)
**2002**	10	4 (40)	106	70 (66)	261	172 (66)	69	42 (61)	446	288 (65)

**2003**	15	7 (47)	135	76 (56)	303	199 (66)	74	50 (68)	527	332 (63)

**2004**	11	4 (36)	96	63 (66)	183	121 (66)	39	27 (69)	329	215 (65)

**2005**	15	7 (47)	65	33 (51)	160	98 (61)	35	22 (63)	275	160 (58)

**2006**	19	10 (53)	50	30 (60)	126	81 (64)	38	25 66)	233	146 (63)

**2007**	7	0 (0)	30	19 (63)	105	69 (66)	34	25 (74)	176	113 (64)

**2008**	6	2 (33)	26	13 (50)	83	53 (64)	49	34 (69)	164	102 (62)

**2009**	8	3 (38)	18	12 (67)	58	40 (69)	31	20 (65)	115	75 (65)

**Total**	91	37 (41)	526	316 (60)	1279	833 (65)	369	245 (66)	2265	1431 (63)

**n = **Number of severe anaemia cases.

**D.P.P **= Number of cases complicated by deep Breathing or Prostration or Profound anaemia (Hb <4 g/dl)

## Discussion

This retrospective study of severe anaemia and transfusion practice has highlighted several important points. A considerable reduction the clinical burden of severe anaemia and requirements for transfusions concurrent with the substantial decline in malaria was noted between 2002 and 2009. Malaria parasitaemia was recorded as the leading cause of anaemia and in the absence of the other complications was associated with a lower mortality than for non-malarial cases of severe anaemia. Severe anaemia complicated by deep breathing, prostration and shock were common and were associated with higher case fatalities. Despite the simultaneous declines in disease burden (malaria and anaemia) and demand on transfusion services we found that this had little effect on the overall case fatality rate of children admitted with severe anaemia, with mortality remaining unchanged at 8% throughout the study period. This was largely explained by the proportion of children with severe anaemia with life threatening complications, which remained constant over the period of study, affecting over 60% requiring transfusion.

These data suggest that the impact of reduced malaria transmission on child morbidity are greater than those measured by changes in malaria admissions alone. This is reinforced by Gerritsen and colleagues study in South Africa (Limpopo) which found a significant reduction of malaria from 1998 to 2007 [15]. The dramatic reduction in the numbers with severe anaemia and consequently the impact on the requirements for blood transfusion are important 'value added' or hidden benefits of malaria control. Quantifying the reduction in transfusion requirements from the year 2002, where over 10% of all children admitted KDH received a blood transfusion (n = 477) to the year 2009 when only 4% received a blood transfusion (n = 181), translated to a 2.6 fold decline. The decrease in blood transfusion requirements, in addition to reducing the risk of transmissible diseases and adverse reactions, has an important health economic impact. Taking an average range of the cost of transfusion per child as between 30-50 U.S. Dollars for voluntary unpaid donors[4], an estimated annual saving of USD 8,880 to 14,800 for this hospital alone.

There are a number of potential explanations why reducing the burden of malaria disease and transfusion requirements did not result in a reduction in case fatality. First, that the proportion with life threatening anaemia did not decline and thus the proportion requiring emergency transfusion remained unchanged. Previous work in Kilifi in 2000 by English and colleagues demonstrated that respiratory distress, prostration and profound anaemia, were the clinical features most associated with death, odds ratios of 4.1 (95% CI 2.2 - 7.4), 7.4 (95% CI 4.2-13.1) and 2.5 (95% CI 1-4 - 4.5) respectively[8]. This data together with that of others indicate no clear association between receipt of transfusion and in-hospital mortality[16]. However, many of the deaths occur on the day of admission, in children awaiting transfusion. A previous report from Siaya, Kenya indicated that transfusion given after the day of admission had no impact on survival[9]. Currently, 20 ml/kg of whole blood (or 10 ml/kg packed cells) is recommended for all levels of anaemia with Hb <6 g/dl[1]. Few data are available on volume of blood received and indicate only modest rises of mean haemoglobin 2.5-3.3 g/dl[8,9,17] and with 25% of children remaining severely anaemic (< 5 g/dL)[8] following initial transfusion. Anecdotal evidence from hospitals suggests that multiple, low volume (20 ml/kg) transfusions are frequently given. Using standard formulae to calculate volume required[18] the current doses prescribed under-treat children with profound anaemia by ~30%. Larger initial transfusion volumes have not been systematically evaluated.

Whilst most groups showed a decline in anaemia and transfusion overtime, there was an increase in children aged 5-12 years in the years 2008-2009. One possible suggestion is that other causes of anaemia were becoming more prominent as a cause of admission in this age group. Unfortunately, in this retrospective study the degree of fine resolution offered by the recorded final diagnosis of severe anaemia does not permit a greater insight into causality.

The effect of introduction of guidelines or protocols that include rationale transfusion practice, restricting blood transfusion use to only those at greatest risk has been examined in several randomized studies in adults, children and newborns. A review of these meta-analyses concludes that restrictive strategies reduce the need for transfusion, in the UK, without increasing morbidity and mortality[19]. Compliance with WHO paediatric guidelines for rationale use of blood transfusion at Kilifi District Hospital is still sub-optimal. Only 55% of patients had anaemia or a degree of severity at admission that would qualify for transfusion if the protocol had been adhered to. The rest of the transfusions were received by children who had moderate, mild or no anaemia at admission. Little change in practice was noted throughout the study period. Finally, it was not possible to differentiate exchange transfusion from transfusion for anaemia in the newborn group. This study had design limitations of that did not allow us to track haemoglobin or markers of severity after admission to know what motivated physicians to transfuse these children. Other studies have shown that strict introduction of strict consensus guidelines was insufficient to change prescribing practice, without regular audit and supervision by senior medical staff [20].

## Conclusions

The consequence of a reduction in the number and proportion of cases admitted with falciparum malaria was a decrease in the burden of severe malarial anaemia and a reduction in the demand for paediatric transfusion. Although the reduced malaria burden leads to a welcome reduction in the transfusion requirement the case fatality for those admitted with severe anaemia remains unacceptably high. The most recent systematic reviews[2,21] indicated the need for formal evaluation of the rationale transfusion policy in a controlled trial. Further research to establish best transfusion practice and identify other treatment strategies aimed at reducing in hospital mortality is essential in this common paediatric condition in sub-Saharan Africa.

## Competing interests

The authors declare that they have no competing interests.

## Authors' contributions

RP did the primary data analysis and prepare the initial and subsequent versions of the manuscript. SA supervised the data analysis and prepartion of the results table and commented on versions of the manuscripts. KM conceived the study, reviewed the data and draft versions of the manuscript. All authors read and approved the final manuscript
