# Changing trends in blood transfusion in children and neonates admitted in Kilifi District Hospital, Kenya

**DOI:** 10.1186/1475-2875-9-364

**Published:** 2010-12-17

**Authors:** Rosalon Pedro, Samuel Akech, Greg Fegan, Kathryn Maitland

**Affiliations:** 1 Kenya Medical Research Institute - Wellcome Trust Programme, PO Box 230, Kilifi, Kenya; 2 Centro de Investigação em Saúde em Angola (CISA), Caxito, Angola; 3 Centre for Clinical Vaccinology and Tropical Medicine, Nuffield Department of Clinical Medicine, University of Oxford, Oxford, UK; 4 Department of Paediatrics and Wellcome Trust Centre for Clinical Tropical Medicine, Faculty of Medicine, Imperial College, Norfolk Place, London, W2 1PG, UK

## Correction

After publication of this work [1], we noted that we inadvertently failed to include the complete list of all coauthors. The full list of authors has now been added and the Authors' contributions and Competing interests section modified accordingly.

## Competing interests

The authors declare that they have no competing interests. GF declares no competing interests.

## Authors' contributions

RP did the primary data analysis and prepare the initial and subsequent versions of the manuscript; SA supervised the data analysis and preparation of the results table and commented on versions of the manuscripts. GF supervised the data analysis and statistical evaluation; KM conceived the study, reviewed the data and draft versions of the manuscript.
